# High-order direct modulation terahertz communications with a wideband time-coding metachip modulator

**DOI:** 10.1126/sciadv.adq8693

**Published:** 2024-11-22

**Authors:** Lan Wang, Jun Yan Dai, Ke Seng Ding, Hong Xin Zeng, Qiang Cheng, Zi Qiang Yang, Ya Xin Zhang, Tie Jun Cui

**Affiliations:** ^1^ School of Physics, University of Electronic Science and Technology of China, Chengdu, China.; ^2^ Zhangjiang Laboratory, Shanghai, China.; ^3^ State Key Laboratory of Millimeter Waves, Southeast University, Nanjing, China.; ^4^ School of Electronic Science and Engineering, University of Electronic Science and Technology of China, Chengdu, China.

## Abstract

Terahertz communication technology based on on-off keying (OOK) direct modulation is vital for sixth-generation communication systems, especially in short-distance and high-rate applications. However, low-order OOK modulation often leads to suboptimal anti-interference capabilities and a heightened demodulation threshold. Here, we propose a high-order direct modulation terahertz communication framework using a wideband time-coding metachip modulator. The modulator leverages the electromagnetic resonance properties within the metaunit structure, with control enabled by gallium arsenide Schottky diodes. By manipulating the timing of voltage pulses applied to these diodes, the equivalent electromagnetic resonance distributions can be precisely regulated in the time domain. This enables independent and accurate control over the amplitude and phase of terahertz harmonics. Leveraging this technique, three high-order modulation schemes—quadrature phase-shift keying, 16-phase-shift keying, and 16-quadrature amplitude modulation— are achieved in a direct modulation and direct detection system, demonstrating the real-time image transmission. The proposed method offers an important way to develop integrated and low-complexity terahertz wireless communication systems.

## Teaser

High-order terahertz communication framework boosts 6G by precisely controlling signal amplitude and phase.

## INTRODUCTION

The demand for wireless capacity is continuously growing with the rapid development of smart terminals and the advent of intelligent sixth-generation (6G) networks (*1*, *2*). As a promising solution for the 6G wireless networks, terahertz wave communication with large capacity and strong directivity plays an essential role in meeting the increasing demands for high-speed and confidential communications (*3*, *4*). Over the past decade, several terahertz wireless communication schemes have been proposed for short-range and high-bandwidth applications (*5*–*8*). Thanks to the development of an external high-speed modulator, the terahertz direct modulation system without analog-to-digital converter (ADC) modules shows unique advantages owing to its simple system architecture and low power consumption (*9*–*11*). Since the metasurface-based terahertz modulator was proposed in 2006 (*12*), the switching speed, insertion loss, and modulation depth have been greatly improved by optimizing the design strategy of metasurface and combining various semiconductor materials (*13*–*16*). Furthermore, terahertz on-chip devices have been reported to achieve high-precision digital phase manipulation through transmission lines with perturbation microstructure units (*17*). Yet, the constraints imposed by fabrication processes and the nonlinear characteristics of materials in the terahertz spectral region have restricted modulators to the fundamental modulation formats, predominantly on-off keying (OOK) used in the terahertz direct modulation/direct detection systems. This limitation is frequently accompanied by suboptimal system anti-interference and a heightened demodulation threshold. The endeavor to achieve a higher-order modulation scheme, facilitating simultaneous and precise control over both amplitude and phase, remains a formidable challenge for terahertz direct modulation communication systems. Meanwhile, the current high-order terahertz communication systems heavily depend on complicated mixing circuits, including high-speed terahertz ADC modules, mixers, and local oscillators (LOs) (*18*–*20*).

Digital coding metasurface with active material has attracted great interest in recent years due to the flexible manipulation of electromagnetic waves (*21*). By combining the signal processing algorithms in the information domain with the real-time programmable elements, a time-domain digital coding metasurface (TDCM) is proposed to digitally manipulate harmonic waves by changing the external biasing with time (*22*–*27*). Based on this, many interesting applications have been reported, especially in the field of wireless communications (*28*–*31*). The real-time manipulations of the electromagnetic responses using TDCMs make it possible to realize space-division multiplexing, frequency-division multiplexing, time-division multiplexing, and space-frequency-division multiplexing in new wireless communication networks (*32*–*35*). Moreover, the control of nonlinear harmonics and the implementation of multiple modulation schemes have been demonstrated (*36*–*39*). The digital coding strategy is commonly integrated into innovative transceiver designs. In comparison with the traditional superheterodyne transmitters, this approach eliminates the need for mixers, thereby reducing the hardware costs.

The time-domain coding method provides efficient approaches for fine regulations of the terahertz waves. The present coding strategy in TDCMs is based on digitally controlling the phase information (*40*, *41*). However, most established terahertz quasi-optical metasurfaces face substantial difficulties, including large-scale circuit processing issues due to their small size and substantial amplitude-phase dependency caused by inherent dispersion (*11*). Therefore, a solution is urgently required to achieve precise control of terahertz waves for the next-generation high-speed wireless communication.

Here, we propose a high-order direct modulation terahertz communication scheme based on a time-coding metachip modulator, achieving accurate control of harmonic waves. We combine the time-coding technology with the metachip so that the transmission coefficient can be periodically switched between two states in real time, allowing us to manipulate the equivalent amplitude and phase distributions of the metachip by adjusting the duty cycle and time delay of the basic symbol waveform. Consequently, the desired harmonic distribution can be generated as needed. This strategy is based on amplitude information rather than phase information, without needing the receiver’s mixer and LO modules. Furthermore, replacing the quasi-optical metasurface with a metachip can promote the system’s miniaturization and integration. Considering the current hardware constraints and cost challenges in terahertz communication systems, precise terahertz harmonic control holds promise as a novel technological approach. To demonstrate this capability, we construct a direct modulation and direct detection system based on the time-coding metachip operating at 340 GHz. By loading different coding sequences onto the OOK metachip modulator, we can directly synthesize the standard constellations at the +first-order harmonic without the need for complex radio frequency circuitry, showing the simplicity and flexibility of the system.

## RESULTS

**Fig. 1.**
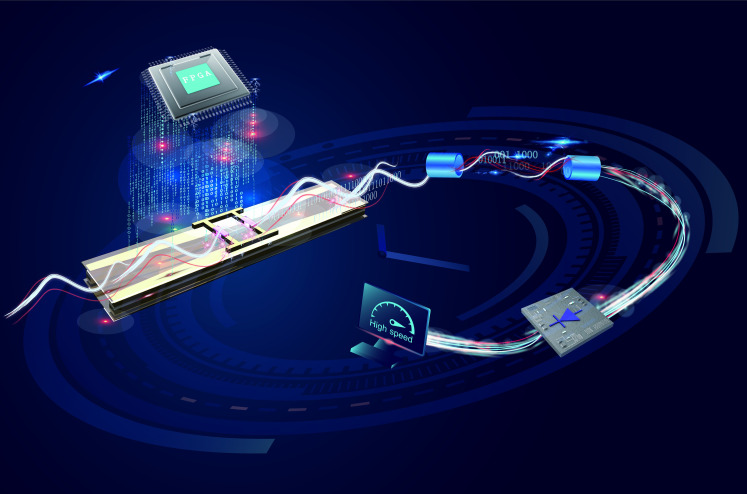
The high-order direct modulation wireless communication system, featuring a metachip modulator and time-coding strategy, reliably propagates terahertz waves through standard rectangular waveguides.

We present to use the time-coding strategy in signal modulations and demodulations of the terahertz communication system, as shown in Fig. 1. The metachip modulator contains on-chip active units embedded with diodes; thus, the transmission amplitude can be switched between two discrete on-off states by controlling the voltage loaded on the diodes. When the control voltage sequence is time-periodically varying with *T*_0_ = 1/*f*_0_, the metachip is capable of converting the input guided wave of frequency *f*_c_ into discrete harmonics. Furthermore, the generated harmonics are distributed at intervals 1/*T*_0_ around the carrier *f*_c_. This approach greatly reduces the complexity of the system hardware, where the transmitter integrates the time-coding metachip with the source, while the receiver requires only a high-speed envelope detector and an amplifier and does not require any complicated terahertz LO and mixer circuits.

### Time-coding strategy based on a terahertz OOK modulator

Generally, the transmission coefficient of the modulator in a symbol period *T*_0_ can be defined as

$$T\left(t\right)=A\left(t\right)\cdot e^{j\Phi \left(t\right)},0\leq t\leq T_{0}$$ (1)

where *A*(*t*) and Φ(*t*) represent the amplitude and phase of the transmission coefficient, respectively. In the traditional direct modulation method, the amplitude and phase of the modulator represent those of the baseband signal, and thus, the waveform remains unchanged in a transmission cycle, such as in the OOK scheme. Such a method simplifies the baseband waveform but limits the modulation order. For example, the amplitude modulator is only suitable for amplitude-shift keying modulation, and the phase modulator is only applicable to phase-shift keying (PSK) modulation. The binary modulator can only realize low-order modulation schemes, such as OOK, binary PSK, and differential PSK. By analogy, high-order modulation schemes, such as quadrature amplitude modulation (QAM), require more complex high-accuracy amplitude-phase joint modulators, which are difficult to design and manufacture in the terahertz band.

**Fig. 2.**
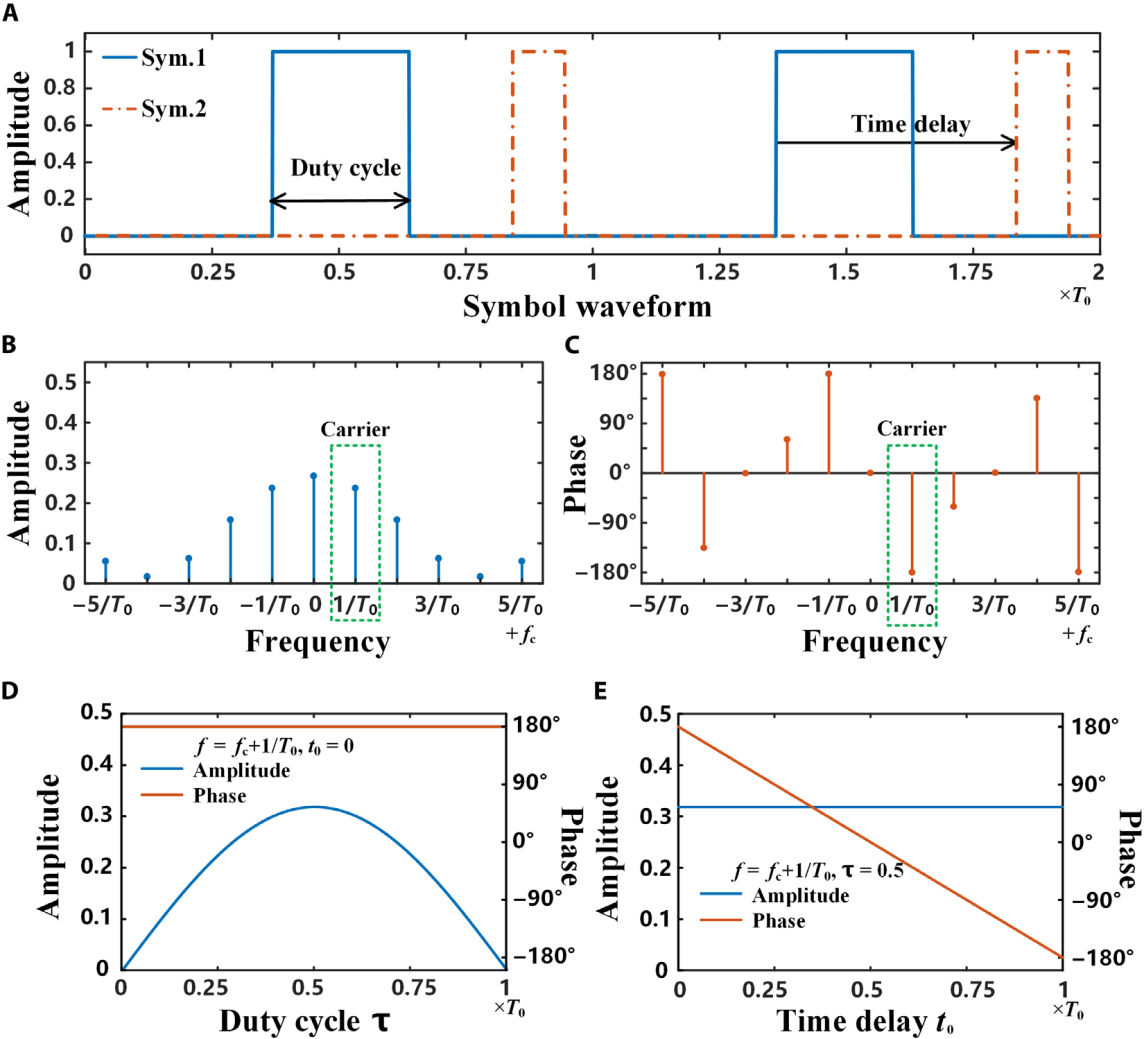
Manipulation principle of the time-coding strategy. (**A**) The diagram of the basic symbol waveform, in which Sym.1 and Sym.2 define different duty cycles and time delays. (**B** and **C**) The harmonic amplitude distribution (B) and phase distribution (C) of the basic symbol waveform after the fast Fourier transform of the symbol period *T*_0_. (**D**) Amplitudes and phases at the +first-order harmonic frequency *f*_c_ + 1/*T*_0_ under different duty cycles. (**E**) Amplitudes and phases at the +first-order harmonic frequency *f*_c_ + 1/*T*_0_ under different time delays.

To solve the dependence problem on the modulator, we introduce the time-coding strategy to generate the harmonics by designing a symbol waveform and modulating the baseband signal with these harmonics (*38*, *40*). Here, we consider a terahertz binary amplitude modulator with a transmission coefficient of either *A*_1_ or *A*_2_, depending on the control signal. Then, we define a basic symbol waveform, as shown in Fig. 2A (*A*_1_ = 0, *A*_2_ = 1), which can be written as follows

$$\begin{array}{c} T(t)=(A_{1}+(A_{2}-A_{1})\sum_{m=-\infty }^{+\infty }\{\varepsilon [t+(m-\frac{1}{2}+\frac{\tau }{2})T_{0}-t_{0}]-\varepsilon [t+(m-\frac{1}{2}-\frac{\tau }{2})T_{0}-t_{0}]\})\\ =\cdot [\varepsilon (t)-\varepsilon (t-T_{0})] \end{array}$$ (2)

in which *m* is a natural number, ε(*t*) is a step function, *t*_0_ is the time delay, and τ is the duty cycle that represents the duration ratio of *A*_2_ in a single cycle. According to the Fourier transform theory, this basic waveform, similar to a square wave, has rich harmonic components. Under the excitation of a terahertz wave with frequency *f*_c_, these harmonic components will be shifted to the terahertz wave frequency. Figure 2 (B and C) shows the corresponding harmonic amplitude and phase distributions of the modulator controlled by the modulation signal (blue solid line in Fig. 2A) in the frequency domain under the excitation of the terahertz wave. The *k*th-order harmonic coefficient is

$$a_{k}=\left\{ \begin{array}{cc} M\cdot A_{2}+\left(1-M\right)\cdot A_{1}, & k=0\\ \left(A_{1}-A_{2}\right)\cdot M\cdot \mathrm{Sa}\left(k\pi M\right)\cdot e^{-\mathrm{jk}\omega_{0}t_{0}}, & k=\pm 1,\pm 2,\pm 3\cdots \end{array}\right.$$ (3)

in which *Sa*(*k*π*M*) = sin(*k*π*M*)/*k*π*M*, and ω_0_ = 2π/*T*_0_ is the harmonic angular frequency.

The amplitudes and phases at the harmonic frequencies can be independently controlled by adjusting the duty cycle and time delay of the basic symbol waveform to realize the signal modulations at the harmonics as the carrier. Take the +first-order harmonic as an example. The amplitudes and phases of the +first-order harmonic under different duty cycles and time delays (*A*_1_ = 0, *A*_2_ = 1) are shown in Fig. 2 (D and E, respectively). We note that the harmonic amplitude has a symmetric distribution about τ = 0.5, which first increases and then decreases with the increasing duty cycle and reaches a peak at τ = 0.5, while the harmonic phase monotonically decreases with the time delay and is cycled within *t*_0_ ∈ [0, *T*_0_]. We remark that the duty cycle amplitude modulation method and the time delay phase modulation method are independent of each other. The theoretical calculation results straightforwardly demonstrate the independence and accuracy of the amplitude and phase modulation methods. These methods can be realized with a terahertz binary amplitude modulator, which lays the foundation to achieve high-order modulation schemes based on time coding.

**Fig. 3.**
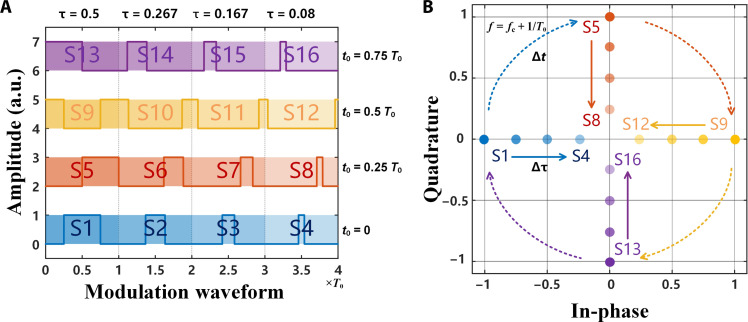
Harmonic modulation principle. (**A**) The symbol waveforms formed by different duty cycles and time delays, in which different color areas represent different transmission symbols (S1 to S16). (**B**) Constellation points of different modulation symbols at the +first-order harmonic frequency *f*_c_ + 1/*T*_0_. a.u., arbitrary units.

In the high-order modulations, obtaining the constellation diagram is a key step. To show the role of time coding more intuitively, Fig. 3A presents the symbol waveforms with different combinations of duty cycle and time delay, forming 16 transmission symbols (S1 to S16), which are represented by different colors. The constellation points of the 16 transmission symbols at the +first-order harmonic are further given in Fig. 3B. The arrow direction of the solid line indicates the amplitude modulation induced by the duty cycle, while the dashed arrow indicates the phase modulation induced by the time delay. Therefore, any constellation distributions can be theoretically synthesized by the time coding, which indicates that any higher-order modulation schemes can be realized by this approach. For example, the duty cycle and time delay combinations required for the symbol waveforms to achieve quadrature phase-shift keying (QPSK), 16-phase-shift keying (16PSK), and 16-quadrature amplitude modulation (16QAM) modulations are listed in Tables 1 to 3, where $\kappa_{A}^{\varphi }$ is defined to represent the amplitude and phase of the constellation point, and $\chi_{\tau }^{t_{0}}$ is defined to indicate the corresponding duty cycle and time delay.

**Table 1.** Relationship between the amplitude and phase of the constellation point, duty cycle, time delay, and transmitted information of the symbol waveform required for the QPSK modulation.

$\kappa_{A}^{\varphi }$	$\kappa_{1}^{45{}^{\circ}}$	$\kappa_{1}^{135{}^{\circ}}$	$\kappa_{1}^{225{}^{\circ}}$	$\kappa_{1}^{315{}^{\circ}}$
$\chi_{\tau }^{t_{0}}$	$\chi_{0.5}^{0.875T_{0}}$	$\chi_{0.5}^{0.625T_{0}}$	$\chi_{0.5}^{0.375T_{0}}$	$\chi_{0.5}^{0.125T_{0}}$
Transmitted information	00	10	11	01

**Table 2.** Relationship between the amplitude and phase of the constellation point, duty cycle, time delay, and transmitted information of the symbol waveform required for the 16PSK modulation.

$\kappa_{A}^{\varphi }$	$\kappa_{1}^{348.75{}^{\circ}}$	$\kappa_{1}^{326.25{}^{\circ}}$	$\kappa_{1}^{303.75{}^{\circ}}$	$\kappa_{1}^{281.25{}^{\circ}}$	$\kappa_{1}^{258.75{}^{\circ}}$	$\kappa_{1}^{236.25{}^{\circ}}$	$\kappa_{1}^{213.75{}^{\circ}}$	$\kappa_{1}^{191.25{}^{\circ}}$
$\chi_{\tau }^{t_{0}}$	$\chi_{0.5}^{0.03125T_{0}}$	$\chi_{0.5}^{0.09375T_{0}}$	$\chi_{0.5}^{0.15625T_{0}}$	$\chi_{0.5}^{0.21875T_{0}}$	$\chi_{0.5}^{0.28125T_{0}}$	$\chi_{0.5}^{0.34375T_{0}}$	$\chi_{0.5}^{0.40625T_{0}}$	$\chi_{0.5}^{0.46875T_{0}}$
Transmitted information	0001	0011	0010	0110	0111	0101	0100	1100
$\kappa_{A}^{\varphi }$	$\kappa_{1}^{168.75{}^{\circ}}$	$\kappa_{1}^{146.25{}^{\circ}}$	$\kappa_{1}^{123.75{}^{\circ}}$	$\kappa_{1}^{101.25{}^{\circ}}$	$\kappa_{1}^{78.75{}^{\circ}}$	$\kappa_{1}^{56.25{}^{\circ}}$	$\kappa_{1}^{33.75{}^{\circ}}$	$\kappa_{1}^{11.25{}^{\circ}}$
$\chi_{\tau }^{t_{0}}$	$\chi_{0.5}^{0.53125T_{0}}$	$\chi_{0.5}^{0.59375T_{0}}$	$\chi_{0.5}^{0.65625T_{0}}$	$\chi_{0.5}^{0.71875T_{0}}$	$\chi_{0.5}^{0.78125T_{0}}$	$\chi_{0.5}^{0.84375T_{0}}$	$\chi_{0.5}^{0.90625T_{0}}$	$\chi_{0.5}^{0.96875T_{0}}$
Transmitted information	1101	1111	1110	1010	1011	1001	1000	0000

**Table 3.** Relationship between the amplitude and phase of the constellation point, duty cycle, time delay, and transmitted information of the symbol waveform required for the 16QAM modulation.

$\kappa_{A}^{\varphi }$	$\kappa_{1}^{225{}^{\circ}}$	$\kappa_{\frac{\sqrt{5}}{3}}^{251.6{}^{\circ}}$	$\kappa_{1}^{315{}^{\circ}}$	$\kappa_{\frac{\sqrt{5}}{3}}^{288.4{}^{\circ}}$	$\kappa_{\frac{\sqrt{5}}{3}}^{198.4{}^{\circ}}$	$\kappa_{\frac{1}{3}}^{225{}^{\circ}}$	$\kappa_{\frac{\sqrt{5}}{3}}^{341.6{}^{\circ}}$	$\kappa_{\frac{1}{3}}^{315{}^{\circ}}$
$\chi_{\tau }^{t_{0}}$	$\chi_{0.5}^{0.375T_{0}}$	$\chi_{0.268}^{0.301T_{0}}$	$\chi_{0.5}^{0.125T_{0}}$	$\chi_{0.268}^{0.199T_{0}}$	$\chi_{0.268}^{0.449T_{0}}$	$\chi_{0.108}^{0.375T_{0}}$	$\chi_{0.268}^{0.051T_{0}}$	$\chi_{0.108}^{0.125T_{0}}$
Transmitted information	0000	0001	0010	0011	0100	0101	0110	0111
$\kappa_{A}^{\varphi }$	$\kappa_{1}^{135{}^{\circ}}$	$\kappa_{\frac{\sqrt{5}}{3}}^{108.4{}^{\circ}}$	$\kappa_{1}^{45{}^{\circ}}$	$\kappa_{\frac{\sqrt{5}}{3}}^{71.6{}^{\circ}}$	$\kappa_{\frac{\sqrt{5}}{3}}^{161.6{}^{\circ}}$	$\kappa_{\frac{1}{3}}^{135{}^{\circ}}$	$\kappa_{\frac{\sqrt{5}}{3}}^{18.4{}^{\circ}}$	$\kappa_{\frac{1}{3}}^{45{}^{\circ}}$
$\chi_{\tau }^{t_{0}}$	$\chi_{0.5}^{0.625T_{0}}$	$\chi_{0.268}^{0.699T_{0}}$	$\chi_{0.5}^{0.875T_{0}}$	$\chi_{0.268}^{0.801T_{0}}$	$\chi_{0.268}^{0.551T_{0}}$	$\chi_{0.108}^{0.625T_{0}}$	$\chi_{0.268}^{0.949T_{0}}$	$\chi_{0.108}^{0.875T_{0}}$
Transmitted information	1000	1001	1010	1011	1100	1101	1110	1111

Note that a large number of unwanted harmonics (non–+first-order harmonics) may be generated during the modulation process, consuming a considerable portion of the input energy. Various digital and analog filtering techniques can be used to eliminate unwanted interference and output a purer modulated signal.

**Fig. 4.**
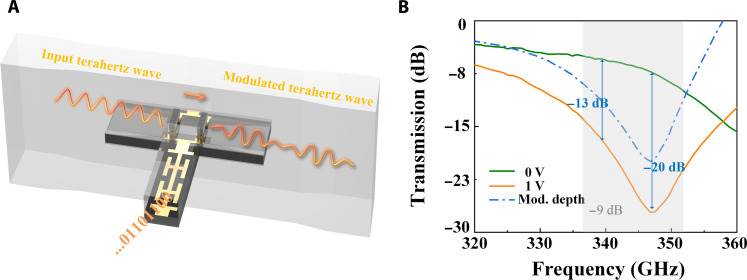
The metachip modulator used for the time-coding strategy. (**A**) The schematic of the modulator. (**B**) The measured transmission coefficients under different voltages. When the applied bias voltage is 0, the metachip exhibits minimal influence on the transmission of the terahertz wave. As the applied voltage is increased to 1 V, the transmission is cut off, attributed to the coupling resonance. The blue dash-dot line illustrates the variation in modulation depth at different frequency points for the two states.

Figure 4A depicts a metachip modulator designed to implement the time-coding strategy. The OOK metachip modulator is composed of active metaunits and a fin-line on the back, referring to the previous work (*42*). A photomicrograph of the metachip and detailed dimensional parameters are provided in fig. S1. The input terahertz wave is guided by the fin-line and transmitted in the quasi-transverse electromagnetic mode on the chip. The time-coding voltage signal is fed through the low-pass filter to control the three-dimensional coupling resonance between the metaunits and the transmitted terahertz wave. Then, the modulated terahertz wave is output by a standard rectangular waveguide port. Figure 4B shows the transmission characteristics of the metachip obtained in the static experiment. Without a direct current bias voltage, we note that the terahertz waves can be transmitted with low loss, around −5.5 dB near 340 GHz. Increasing the applied voltage to 1 V, the electrons flow in the metaunit on both sides of the gallium arsenide Schottky diodes (*43*, *44*), and the coupling resonance between the metaunit and fin-line is formed, which can cut off the terahertz wave at the position of the metaunit and block the transmission of most energies. The modulation depth can reach more than −9 dB in the 352- to 366-GHz range, including −13 dB at 340 GHz, and the maximum modulation depth can reach −20 dB with an insertion loss of only −7 dB, as shown in the gray area in Fig. 4B. The experimental results demonstrate that the packaged metachip modulator achieves outstanding switching effect in a wide frequency range around 340 GHz.

**Fig. 5.**
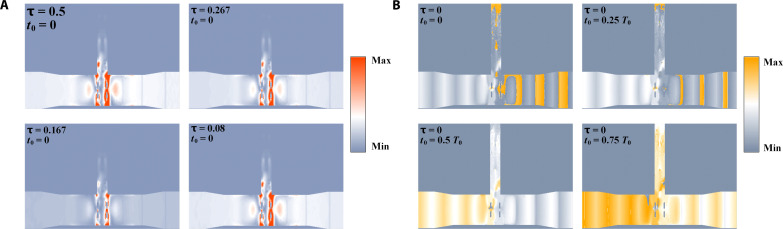
The simulated mode distributions at the +first-order harmonic. (**A**) The equivalent amplitude distributions under different duty cycles with the time delay *t*_0_ = 0. The duty cycle only affects the harmonic amplitude distribution, while the phase distribution remains consistent. (**B**) The equivalent phase distributions under different time delays with the duty cycle τ = 0.5. The phase undergoes a gradual transformation as the time delay ranges from 0 to 0.75 *T*_0_, while the amplitude remains constant.

To explain the mechanism of high-order amplitude and phase conversions by the time coding in the OOK modulator, field distributions of the equivalent electromagnetic mode of the +first-order harmonic excited by the time-coding voltage signal are presented (see Materials and Methods), as shown in Fig. 5 (A and B). First, we examine the electric fields of the metachip in both on and off states. By subjecting the 1-GHz modulation signal to the Fourier transform, we extract the amplitude and phase distributions at the +first-order harmonic under various duty ratios and time delays. The effect of the duty cycle on the amplitude distribution is notable, with the field strength reaching its peak at τ = 0.5 and diminishing as the duty cycle decreases from 0.5 to 0.08. Specifically, the duty cycle only affects the harmonic amplitude distribution, while the phase distribution remains consistent. Correspondingly, the phase distribution can be manipulated by adjusting the time delay. As shown in Fig. 5B, the phase undergoes a gradual transformation as the time delay ranges from 0 to 0.75 *T*_0_, while the amplitude remains constant. Thus, by manipulating the duty cycle and time delay of the OOK signal in the time domain, we achieve independent and precise modulations of both the amplitude and phase of the terahertz wave. These results confirm that we can obtain high-order QAM modulations based on OOK by controlling the time-domain duty cycle and time delay independently and simultaneously.

### Implementation of the wireless communication system

**Fig. 6.**
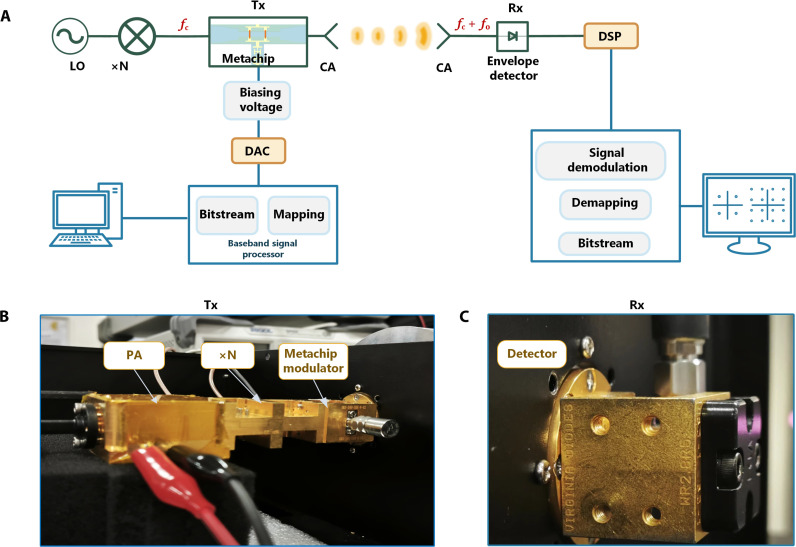
Experimental setup. (**A**) Block diagram of the experimental setup. In the transmitting terminal, the transmitted bitstreams are mapped into the coding sequences with the corresponding duty cycles and time delays, and then are loaded to the metachip modulator. In the receiving terminal, the received signal at *f*_c_ + *f*_0_ (*f*_c_ = 340 GHz and *f*_0_ = 1.25 GHz) is demapped to the bitstreams. DSP, digital signal processing. (**B**) Photograph of the transmitter, including the power amplifier (PA), the frequency multiplier, and the metachip modulator. (**C**) Photograph of the detector. In the above panels, Tx represents the transmitter, and Rx represents the receiver. N, the frequency multiplication integer; CA, Cassette Antenna; DAC, Digital-to-Analog Converter.

To demonstrate the viability of the generalized time-coding strategy, we constructed a terahertz direct modulation and direct detection wireless communication system at *f*_c_ = 340 GHz based on the metachip modulator. Figure 6A delineates the block diagram of the terahertz wireless communication system. The system’s effectiveness is attributed to the precise control of $\kappa_{A}^{\varphi }$ and the robustness of the coding strategy, facilitating the successful implementation of 16QAM at the +first-order harmonic. To explicate the comprehensive communication process, the QPSK modulation serves as an illustrative case. At the transmitter end, the bitstreams (e.g., 100110…) representing an image undergo systematic mapping onto the corresponding coding sequences of the metachip. Specifically, 00 corresponds to $\chi_{0.5}^{0.875T_{0}}$, 10 corresponds to $\chi_{0.5}^{0.625T_{0}}$, 11 corresponds to $\chi_{0.5}^{0.375T_{0}}$, and 01 corresponds to $\chi_{0.5}^{0.125T_{0}}$. Subsequently, the digital signal is loaded onto the metachip through the digital input module. At the receiver end, the received signal undergoes digital signal processing, where the recovered signal is transformed into the frequency domain through a fast Fourier transform. The detection of amplitude (*A*) and phase (φ) for each message symbol culminates in the successful recovery of the corresponding transmitted information.

As illustrated in Fig. 6B, the terahertz transmitter setup includes an LO to generate a signal at 14.167 GHz with a power of 6 dBm, which is then fed into a chain consisting of a multiplier and a power amplifier to produce a 340-GHz signal serving as the terahertz carrier. The time-coding information is loaded onto the metachip modulator, and the terahertz wave carrying the +first-order harmonic (341.25 GHz) digital information is radiated into free space through an antenna. At the receiver shown in Fig. 6C, a high-speed Schottky diode envelope detector is used for reception. The detailed parameters for each part of the terahertz system are provided in Supplementary Text, section S1.

**Fig. 7.**
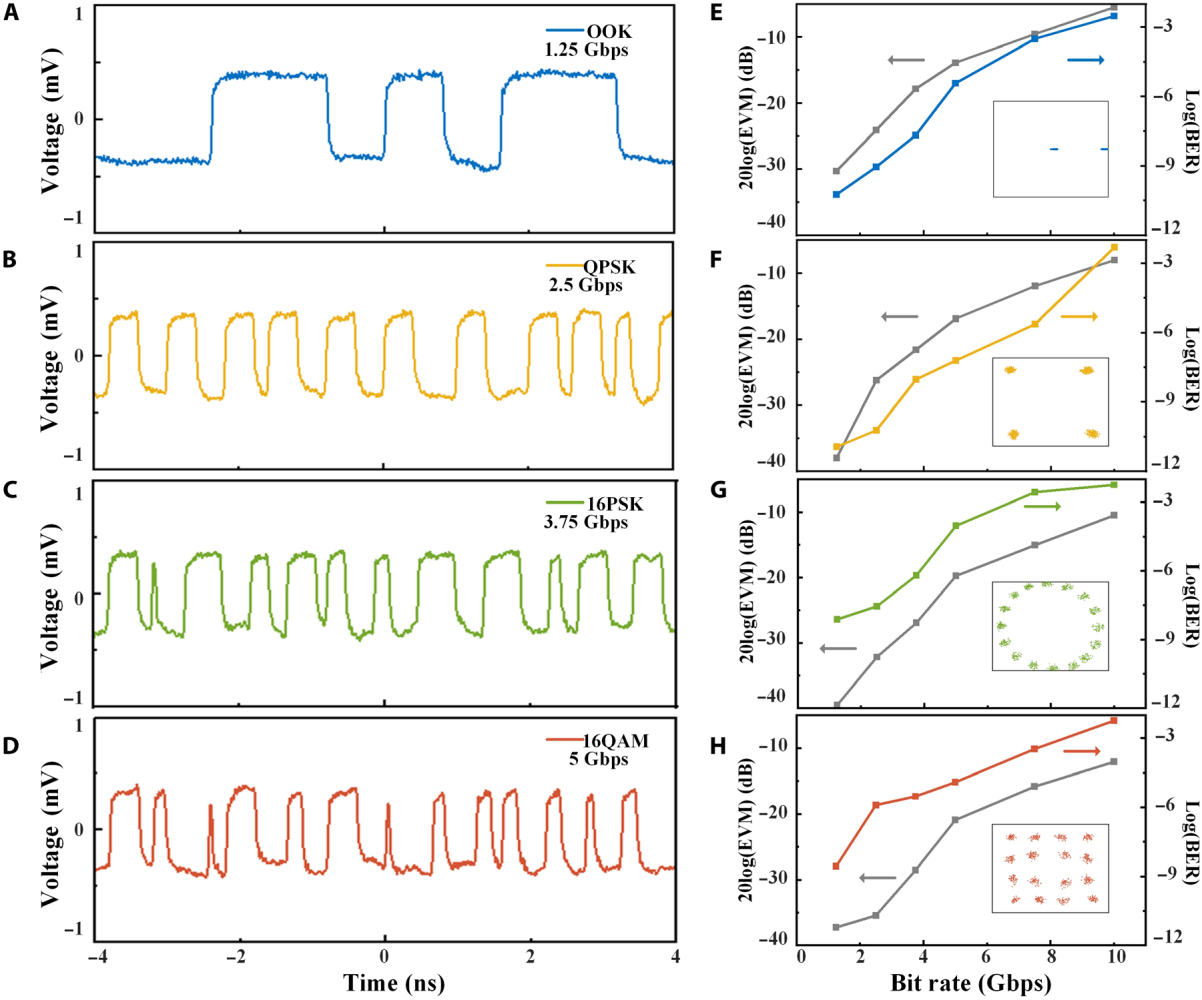
The experimental results of various modulation schemes. (**A** to **D**) Waveforms of the demodulated signals of OOK, QPSK, 16PSK, and 16QAM at bit rates of 1.25, 2.5, 3.75, and 5 Gbps, respectively. (**E** to **H**) Error vector magnitude (EVM) and bit error rate (BER) as the functions of the bit rate for different modulation schemes. The inset shows the OOK, QPSK, 16PSK, and 16QAM constellation diagrams corresponding to (A) to (D).

To validate the potentials of the high-speed and high-order modulations using the terahertz time-coding metachip modulator, we used an arbitrary waveform generator (Keysight M8194A) to drive the metachip system. At the receiver end, the waveforms were recorded using a real-time oscilloscope (Agilent DSO-X 93204A) with a sampling rate of 80 GSa/s. As shown in Fig. 7, a series of time-varying coding sequences characterized by distinct duty cycles and time delays corresponding to various modulation formats (refer to Tables 1 to 3) is loaded in the metachip at varying bit rates. Figure 7 (A to D) shows the received waveforms of different modulation formats (OOK, QPSK, 16PSK, and 16QAM) at the corresponding bit rates of 1.25, 2.5, 3.75, and 5 Gbps, respectively. The constellation diagram, bit error rate (BER), and error vector magnitude (EVM) at the +first-order harmonic (341.25 GHz) are extracted from the collected data (see Materials and Methods), as illustrated in Fig. 7 (E to H). We provide a summary of BERs and EVMs as the functions of bit rate to evaluate the communication performance across different modulation schemes. Notably, with an increase in bit rate, there is a slight drift in constellation points, coupled with an elevation in both BER and EVM. This phenomenon can be attributed to the distortion of the modulated signal induced by the limited bandwidth of the control circuit.

To further demonstrate the viability of the time-coding strategy, we conducted real-time demodulation of the constellation diagram and made experiments of image transmissions. Specifically, the terahertz wave carrying the digital information was demodulated at the receiver using the oscilloscope, revealing the demodulated 1.6-Gbps QPSK constellation diagram. The detailed experimental setup and results are provided in fig. S2. In addition, we conducted real-time image transmissions over a distance of 2 m, and the image was successfully recovered at the receiver using the QPSK modulation scheme. This result underscores the effectiveness of the proposed communication system. Detailed experimental setup and video are available in fig. S3 and movie S1.

## DISCUSSION

With an increasing number of cores being integrated into a single chip, the on-chip wireless communication has become a critical issue, especially considering that under 35-nm technology, interconnect Resistor-Capacitor (RC) delay is 100 times higher than Metal-Oxide-Semiconductor Field-Effect Transistor (MOSFET) response time. Terahertz wireless communication presents a potential solution for communication between multicore chip processors, helping to avoid information exchange between cores becoming a bottleneck for computational speed. The design of a system-on-chip (SoC) architecture must consider several requirements, including the simplified system architecture, low-power design strategies, certain programmability, and cost optimization (*45*–*47*). The end-to-end solution that we propose, which combines time coding with direct modulation and detection, offers a potentially promising approach for SoC applications. Leveraging complementary metal-oxide semiconductor technology, this approach is expected to evolve toward higher integration levels and eventually achieve full on-chip integration. We have conducted a detailed comparison of terahertz systems around 300 GHz in Supplementary Text, section S2.

The proposed strategy based on the time-coding metachip promotes the miniaturization and integration of the system, supporting various modulation schemes. We only used a metachip to achieve independent and precise controls over the amplitude and phase of the terahertz harmonic. By manipulating the duty cycle and time delay, we gain control over the equivalent resonance distributions of the on-chip metastructure, facilitating high-order modulation conversions of the terahertz harmonic orthogonal amplitudes and phases. Last, we constructed the terahertz time-coding direct modulation communication system and implemented multiple modulation schemes, including QPSK, 16PSK, and 16QAM at 341.25 GHz, with a bit rate of up to 5 Gbps. This strategy eliminates the need for the receiver’s mixer and LO modules, which greatly reduces the complexity of system architecture and hardware. The proposed method provides an important technique for developing high-performance, low-cost, and low-complexity terahertz wireless communication systems, which could also become potential competitive solutions for the wireless network-on-chip.

## MATERIALS AND METHODS

### Details of simulations

The equivalent amplitude and phase distributions of the terahertz metachip are presented to demonstrate the high-order amplitude-phase conversions achieved by the time-coding voltage signals in the modulator. Figure S4 shows the electric-field amplitude and phase for the terahertz wave transmission across the chip with the Schottky diodes in the on and off states (applied voltage is 0 and 1 V, respectively). The modulator is designed using CST Studio Suite, and figs. S5 and S6 show the detailed simulation setup. Considering the modulation with a 1-GHz signal, the complex electric-field expressions with various time-coding sequences can be obtained by combining Eq. 3. Through the Fourier transform operation, the equivalent electric-field amplitude and phase distributions of the terahertz on-chip modulator at the +first-order harmonics under different duty ratios and delay coding sequences can be obtained.

### BER and EVM

BER and EVM are used to judge the quality of the encoded signals because the limited data length did not allow for a reliable measurement of the BER. BER is a measure of the accuracy of data transmission within a specified time, which is usually defined as the number of bits received with errors compared to the total number of transmitted bits. EVM describes the error vector of the offset between the actual received signal vector and the ideal transmitted vector in the IQ constellation plane. The EVM is defined as follows (*48*)

$$\text{EVM}=\frac{\sigma_{\text{err}}}{|E_{t}|}$$

$$\sigma_{\text{err}}^{2}=\frac{1}{N}\sum_{i=1}^{1}{|E_{\text{err},i}|}^{2}$$

$$E_{\text{err},i}=E_{r,i}-E_{t,i}$$

in which *N* represents the total number of transferred data, and *E*_err,*i*_, *E*_*r*,*i*_, and *E*_*t*,*i*_ are the *i*th error vector, the *i*th received signal vector, and the *i*th ideal constellation vector, respectively.

## Supplementary Materials

### The PDF file includes:

Supplementary Text Figs. S1 to S6 Table S1 Legend for movie S1 References
