# Supplementary material for: High-order direct modulation terahertz communications with a wideband time-coding metachip modulator
Source: Sci Adv. 2024 Nov 22;10(47):eadq8693. doi: 10.1126/sciadv.adq8693 (PMC11801051; doi:10.1126/sciadv.adq8693)
Supplement: Supplementary file 1 — Supplementary Text Figs. S1 to S6 Table S1 Legend for movie S1 References [file sciadv.adq8693_sm.pdf]

Science Advances
AAAS

Supplementary Materials for

High-order direct modulation terahertz communications with a wideband time-coding metachip modulator

Lan Wang *et al.*

Corresponding author: Ya Xin Zhang, zhangyaxin@uestc.edu.cn; Tie Jun Cui, tjcui@seu.edu.cn

Sci. Adv. **10**, eadq8693 (2024)
DOI: 10.1126/sciadv.adq8693

The PDF file includes:

Supplementary Text
Table S1
Figs. S1 to S6
Legend for movie S1
References

Other Supplementary Material for this manuscript includes the following:

Movie S1

Supplementary Text

Section S1: The terahertz RF system contains the following components

1) 340 GHz signal generation

The 340 GHz signal generation link comprises a 14.167 GHz local oscillator, an amplifier, and a 24 times frequency multiplication link. The 24 multipliers and amplifier link consist of six key components: 6 multipliers, the power amplifier module, the power divider, two doublers, the power synthesizer, and two additional doublers. In the frequency range from 335 GHz to 345 GHz, the output power of the frequency multiplication link exceeds 20 mW.

2) Detector

The receiver's detector employs a zero-bias detector (VDI, WR2.8ZBD). Its operating frequency range is 260-400 GHz, with the maximum available response rate of 40 GHz.

3) Meta-chip modulator

The meta-chip modulator is composed of active meta-units and a fin-line on the back, as shown in Supplementary Fig. S1. The chip size is 1.480×0.29 mm^2. The working frequency is 340 GHz with a wavelength of approximately 0.882 mm. The meta-component length is about one-tenth of the wavelength, categorizing it in the electrically small size range. The bias voltage of the modulator is 0.7 V, and the maximum input power is 10 mW. Therefore, to ensure the normal operation of the modulator, an additional attenuator needs to be added at the frontend to ensure the proper functions of the modulator.

4) Antenna

In order to compensate for the path loss and atmospheric absorption loss in the terahertz wireless transmission, we employed high gain Cassegrain antenna or Lens antenna to provide a gain of 45 dBi. Precise alignment of the transmitter and receiver antennas can be achieved by lightweight azimuth-elevation gimbals (with a rotation accuracy of 0.1°). With the fixed transmitter power, the alignment was confirmed when adjusting the antennas' horizontal and pitch angles, resulting in the maximum received power.

5) Wireless link power consumption analysis

We conducted a link budget analysis of the wireless communication system. Taking a desktop short-range transmission system (see Supplementary Fig. S2) as an example, the power input to the OOK modulator at the transmitter is 6.53 dBm, and the output power from the modulator is 1.14 dBm. Subsequently, the signal is radiated into free space via a Cassegrain antenna with a 45 dBi transmission gain. According to the Friis transmission formula,

$$FSPL = 20\log\left(4\pi d f_c / c\right)$$

The free space path loss (FSPL) is approximately 78dB. However, since the distance between the transmitting and receiving antennas is only 60cm, and the transmission beamwidth of the antenna is only 0.3 degrees, this results in decreased efficiency in signal radiation and capture. Hence, the output power at the receiving antenna drops to -10 dBm.

Section S2: End-to-end comparison of terahertz systems around 300 GHz

We have conducted a detailed comparison of terahertz systems around 300 GHz in Table S1, evaluating key indicators such as power consumption, cost, and footprint between the proposed solution and the most advanced existing end-to-end solutions. In systems operating above 300 GHz, higher-order modulation is typically achieved using a superheterodyne architecture. Such receivers usually require a local oscillator (LO) and a mixer. For instance, in the III-V compound semiconductor process, the mixer and LO power amplifier fabricated with the 80-nm InP-HEMT process, as referenced in (*50*), can cost several thousand dollars. However, in the direct modulation and detection architecture we employ, there is no need for down conversion at the receiver, eliminating the need for an LO, thus offering advantages in terms of cost and footprint.

While CMOS technology offers high integration, low cost, and low power consumption, its THz system is limited in transmission distance due to the currently low signal power at THz frequencies. It is anticipated that the time-coding combined with direct modulation and detection architecture we propose, when implemented in CMOS technology, can further reduce the system chip size.

Terahertz photonic-assisted wireless systems, especially those employing UTC-PDs and optical mixing technology, generally show higher power consumption. This is primarily due to the low conversion efficiency of UTC-PD and the high power output of continuous wave (CW) laser sources required. For instance, supplier quotes indicate that a 280-380 GHz UTC-PD from Japanese NTT Corporation costs over $34,000 each.

Table S1. End-to-end comparison of terahertz systems around 300 GHz

	Frequency (GHz)	TRX architecture	Data rate (Gbps)	Service stream transmission	Distance	Power Consumption*	Footprint**	Cost***	Ref
InP-HEMT	300	Heterodyne	120	NO	9.8m	31.05W(RF)<br>50W(AWG)	64.23cm^2(RF)<br>2475cm^2(AWG)	$117200 (RF)<br>$148812 (AWG)	[20]
InGaAs-mHEMT	300	Superheterodyne	60	NO	10m	34.6W(RF)<br>50W(AWG)	68cm^2(RF)<br>2475(AWG)	$111428(RF)<br>$148812(AWG)	[49]
InGaAs mHEMT	300	Superheterodyne	32	NO	0m	29.45W(RF)<br>180W(AWG)	138cm^2(RF)<br>2475 cm^2(AWG)	$122900(RF)<br>$148812(AWG)	[50]
28nm Si CMOS	390	Coherent detection	6	No	Back to back	1.1W(Chip)<br>50W(AWG)	56.04cm^2(RF)<br>2475cm^2(AWG)	/	[51]
65nm Si CMOS	300	Coherent detection	34	No	4cm	0.407W(Chip)<br>60W(AWG)	143.75 cm^2(RF)<br>2475 cm^2(AWG)	$31631(RF)<br>$148812(AWG)	[52]
UTC-PD	408	Heterodyne	131	NO	10.7m	101.507W(RF)<br>50W(AWG)	1687.5cm^2(RF)<br>2475 cm^2(AWG)	$46800(RF)<br>$148812(AWG)	[53]
UTC-PD	300	Coherent detection	48	Yes	3cm	201.014W(RF)<br>12W(SDI)	2573.5cm^2(RF)<br>225cm^2(SDI)	$96600(RF)<br>$1000(SDI)	[54]
UTC-PD	300	Coherent detection	115	NO	110m	111.007W(RF)<br>50W(AWG)	1707.5cm^2(RF)<br>2475 cm^2(AWG)	$84800(RF)<br>$148812(AWG)	[8]
Schottky Diode	341.5	Direct modulation /detection	5/1.6	Yes	2m/60cm	14.68W(RF)<br>35W(FPGA)	78.82cm^2(RF)<br>384cm^2(FPGA)	$26860(RF)<br>$1066(FPGA)	This work

*The power consumption of all RF components in the link has been calculated, with the power consumption of the AWG/FPGA listed separately.
**The footprint refers to the area occupied by the device after the chip has been packaged.
***The prices are estimated based on publicly available information or supplier quotes; in large-scale commercial use, the prices are expected to be lower.

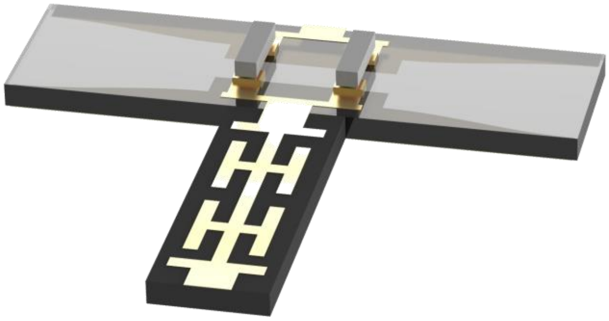

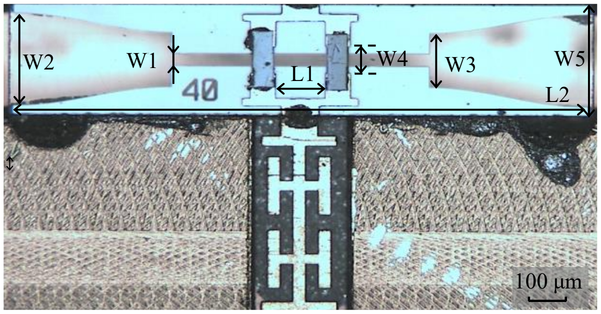

Labels	Dimensions (mm)	Labels	Dimensions (mm)
L1	0.13	W3	0.246
L2	1.48	W4	0.08
W1	0.04	W5	0.346
W2	0.29	H	0.05

Supplementary Fig. S1. Photomicrograph of the meta-chip and detailed dimensional parameters. H is the chip thickness.

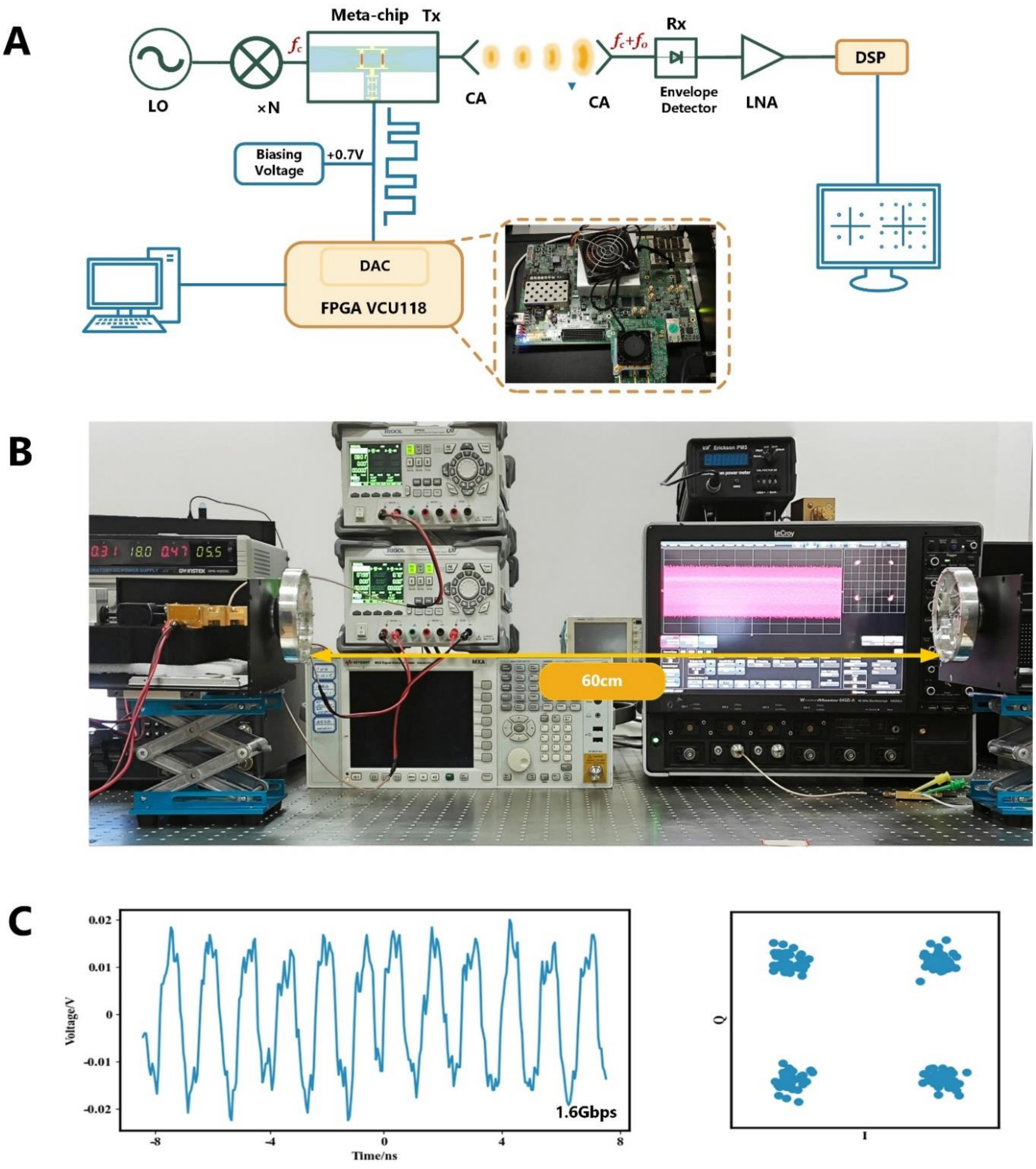
A
Meta-chip
Tx
Rx
LO
×N
f_c
CA
CA
f_c+f_0
Envelope Detector
LNA
DSP
Biasing Voltage
+0.7V
DAC
FPGA VCU118
B
60cm
C
Voltage/V
Time/ns
1.6Gbps
0.02
0.01
0
-0.01
-0.02
-8
-4
0
4
8
Q
I

Supplementary Fig. S2. Experimental setup and results. (**A**) The modulator loads the data signal with a bandwidth of B onto the terahertz carrier at a frequency of fc. The 340 GHz terahertz carrier is generated by a local oscillator (LO) and multiplier amplifier chain. Subsequently, the modulated terahertz signal is radiated and received via a pair of Cassegrain antennas (CA). The transmission distance amounts to 60 cm. After the transmitted signal was received by the high-speed Schottky diode envelope detector, it was amplified by a low-noise amplifier (LNA, AT-BBLF-0043-2720C) with a gain of 25 dB before being connected to the oscilloscope (WaveMaster845Zi-A). The oscilloscope enabled real-time demodulation of the time-coded waveform and constellation diagram. (**B**) Real-time transmission photograph of the desktop system. (**C**) The oscilloscope captured the 1.6 Gbps time-coding waveform and real-time demodulated QPSK constellation diagram

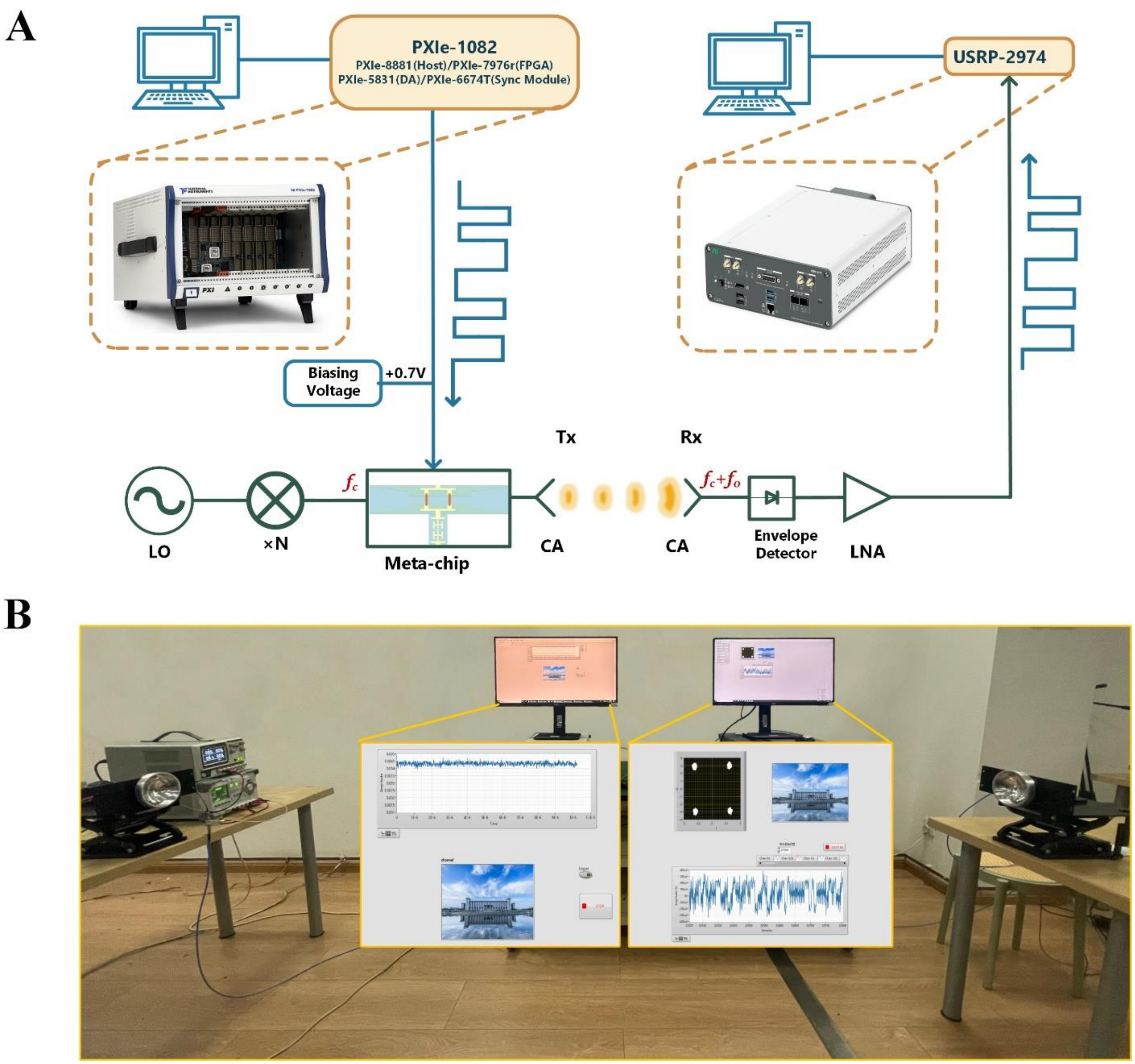

Supplementary Fig. S3. Experimental setup and results. (**A**) Experimental setup. The inset shows an actual transmitted and demodulated image. The transmitter includes a host computer controller, an FPGA module (PXIe-7976r) for baseband signal processing, a DA module (PXIe-

5831), a system synchronization clock module (PXIe-6674T), and a frequency multiplication link providing a 340 GHz carrier, along with a terahertz direct modulation chip. The receiver includes a software-defined radio (USRP-2974) for signal demodulation, as well as an envelope detector and a low-noise amplifier for detecting and amplifying the wireless signal. The transmitted image data is converted into a time-coding signal by a PXIe module, which drives a terahertz meta-chip modulator. This signal is then radiated into free space through a Cassegrain antenna. Upon reception, the signal passes through a detector and a low-noise amplifier (LNA) before being input into the receiver for real-time demodulation and image data recovery. (**B**) Photograph of the real-time image transmission test system with a transmission distance of 2 m. Limited by the sampling rate of the FPGA, the modulation format was set to QPSK, with a real-time transmission rate of 10 Mbps.

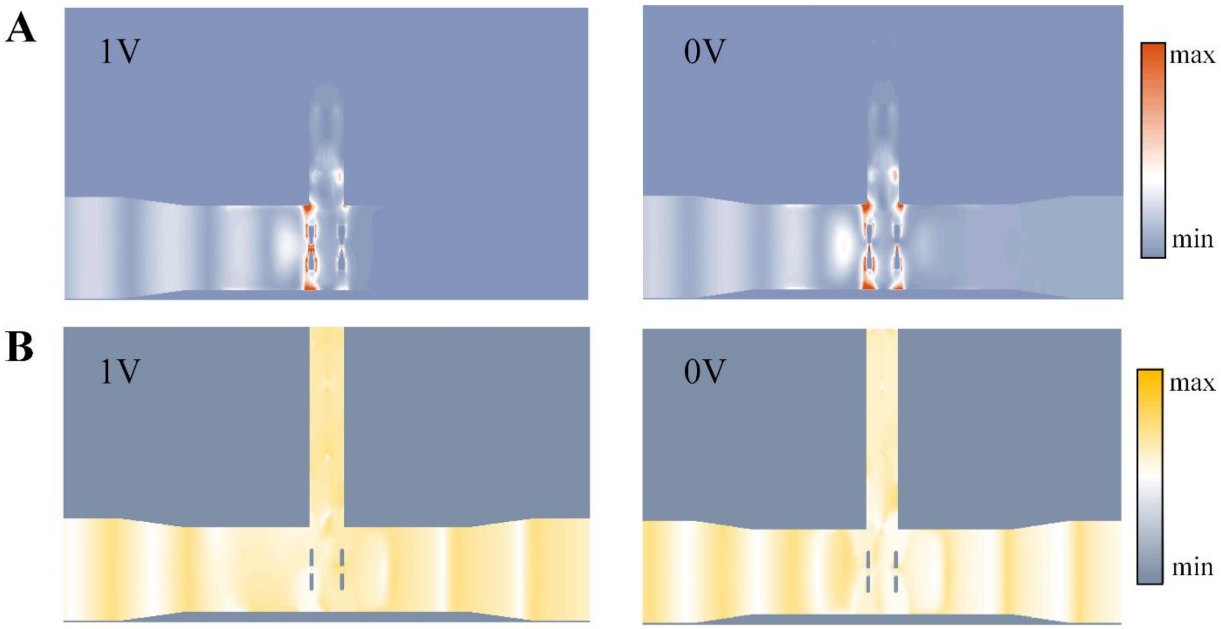

Supplementary Fig. S4. The simulated mode distributions at 340 GHz. (A) The amplitude distribution on the chip under the two states of the diode. **(B)** The phase distribution on the chip under the two states of the diode. When 0 V is applied to the diode, the terahertz wave can pass through the finline with almost no attenuation; at 1 V, the terahertz wave is blocked, preventing the energy from passing through.

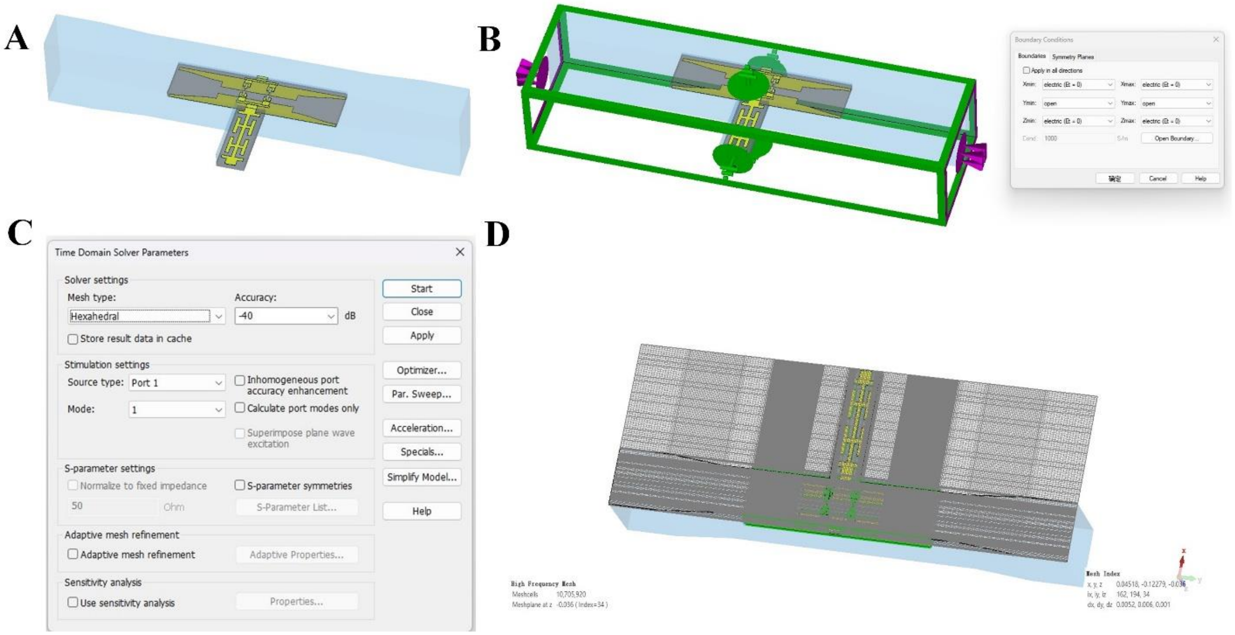

Supplementary Fig. S5. The simulation setup. (**A**) The simulation model. (**B**) Time domain solver settings. (**C**) Boundary conditions. Since electromagnetic waves propagate within the waveguide and the waveguide walls are made of highly conductive metal, only a vacuum cavity needs to be set. The "background" is set as a perfect electric conductor (PEC). The boundaries outside the propagation direction are set as open, and the boundaries perpendicular to the propagation direction are set as electric boundaries (Et=0), effectively simulating the waveguide. (**D**) Mesh settings. Both hexahedral mesh and local mesh refinement are used simultaneously to improve the accuracy of the simulation results. When the difference between the results of consecutive iterations falls within the specified tolerance, it indicates that the simulation has converged and that the mesh has achieved the required precision for simulation analysis.

A

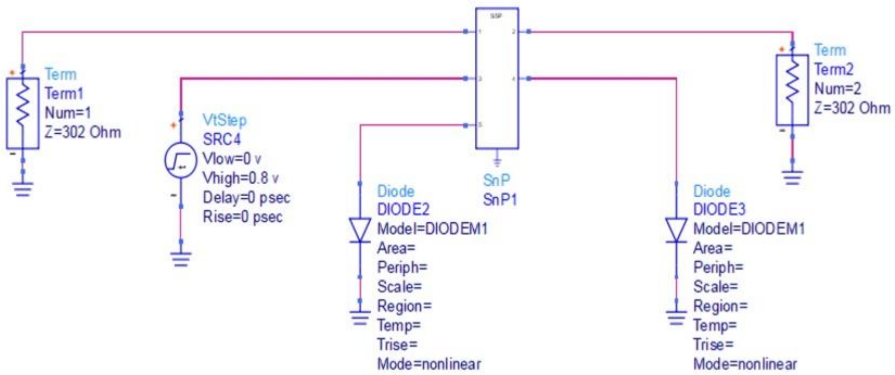

B

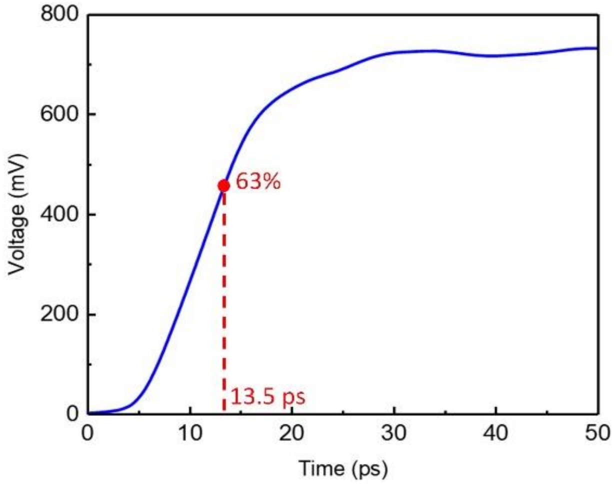

Supplementary Fig. S6. The RC time constant. (**A**) The Simulation model of THz electronic circuit. (**B**) Simulation results of charging curve. The RC time constant is equal to the time for the voltage of the THz modulator to reach 1/e (63%) of the maximum value. Therefore, the RC time constant is determined to be τ=13.5 ps. The smaller the time constant, the faster the system responds, which means that the rising edge time will be shorter, and hence higher signal bandwidth that can be supported.

Movie S1.

High-order direct-modulation terahertz communication system

REFERENCES AND NOTES

1. H. Yang, A. Alphones, Z. Xiong, D. Niyato, J. Zhao, K. Wu, Artificial-intelligence-enabled intelligent 6G networks. *IEEE Netw.* **34**, 272–280 (2020).

2. S. Dang, O. Amin, B. Shihada, M.-S. Alouini, What should 6G be? *Nat. Electron.* **3**, 20–29 (2020).

3. T. Nagatsuma, G. Ducournau, C. C. Renaud, Advances in terahertz communications accelerated by photonics. *Nat. Photonics* **10**, 371–379 (2016).

4. J. Ma, R. Shrestha, J. Adelberg, C.-Y. Yeh, Z. Hossain, E. Knightly, J. M. Jornet, D. M. Mittleman, Security and eavesdropping in terahertz wireless links. *Nature* **563**, 89–93 (2018).

5. L. Moeller, J. Federici, K. Su, 2.5 Gbit/s duobinary signalling with narrow bandwidth 0.625 terahertz source. *Electron. Lett.* **47**, 856–858 (2011).

6. Y. Yang, Y. Yamagami, X. Yu, P. Pitchappa, J. Webber, B. Zhang, M. Fujita, T. Nagatsuma, R. Singh, Terahertz topological photonics for on-chip communication. *Nat. Photonics* **14**, 446–451 (2020).

7. C. Jastrow, S. Priebe, B. Spitschan, J. Hartmann, M. Jacob, T. Kürner, T. Schrader, T. Kleine-Ostmann, Wireless digital data transmission at 300 GHz. *Electron. Lett.* **46**, 661–663 (2010).

8. T. Harter, C. Füllner, J. N. Kemal, S. Ummethala, J. L. Steinmann, M. Brosi, J. L. Hesler, E. Bründermann, A.-S. Müller, W. Freude, S. Randel, C. Koos, Generalized Kramers–Kronig receiver for coherent terahertz communications. *Nat. Photonics* **14**, 601–606 (2020).

9. Y. Liang, C. C. Boon, H. C. Zhang, X.-L. Tang, Q. Zhang, H. Yu, A 13.5-Gb/s 140-GHz silicon redriver exploiting metadevices for short-range OOK communications. *IEEE Trans. Microwave Theory Tech.* **70**, 239–253 (2021).

10. C. Yi, S. H. Choi, M. Urteaga, M. Kim, 20-Gb/s ON–OFF-keying modulators using 0.25-μm InP DHBT switches at 290 GHz. *IEEE Microw. Wirel. Compon. Lett.* **29**, 360–362 (2019).

11. Y. Zhang, S. Qiao, S. Liang, Z. Wu, Z. Yang, Z. Feng, H. Sun, Y. Zhou, L. Sun, Z. Chen, X. Zou, B. Zhang, J. Hu, S. Li, Q. Chen, L. Li, G. Xu, Y. Zhao, S. Liu, Gbps terahertz external modulator based on a composite metamaterial with a double-channel heterostructure. *Nano Lett.* **15**, 3501–3506 (2015).

12. H.-T. Chen, W. J. Padilla, J. M. O. Zide, A. C. Gossard, A. J. Taylor, R. D. Averitt, Active terahertz metamaterial devices. *Nature* **444**, 597–600 (2006).

13. M. Mittendorff, S. Li, T. E. Murphy, Graphene-based waveguide-integrated terahertz modulator. *ACS Photonics* **4**, 316–321 (2017).

14. Y. Zhao, L. Wang, Y. Zhang, S. Qiao, S. Liang, T. Zhou, X. Zhang, X. Guo, Z. Feng, F. Lan, Z. Chen, X. Yang, Z. Yang, High-speed efficient terahertz modulation based on tunable collective-individual state conversion within an active 3 nm two-dimensional electron gas metasurface. *Nano Lett.* **19**, 7588–7597 (2019).

15. P. K. Singh, S. Sonkusale, High speed terahertz modulator on the chip based on tunable terahertz slot waveguide. *Sci. Rep.* **7**, 40933 (2017).

16. L. Cong, Y. K. Srivastava, H. Zhang, X. Zhang, J. Han, R. Singh, All-optical active THz metasurfaces for ultrafast polarization switching and dynamic beam splitting. *Light Sci. Appl.* **7**, 28 (2018).

17. H. Zeng, H. Liang, Y. Zhang, L. Wang, S. Liang, S. Gong, Z. Li, Z. Yang, X. Zhang, F. Lan, Z. Feng, Y. Gong, Z. Yang, D. M. Mittleman, High-precision digital terahertz phase manipulation within a multichannel field perturbation coding chip. *Nat. Photonics* **15**, 751–757(2021).

18. P. Rodriguez-Vazquez, J. Grzyb, B. Heinemann, U. R. Pfeiffer, A QPSK 110-Gb/s polarization-diversity MIMO wireless link with a 220–255 GHz tunable LO in a SiGe HBT technology. *IEEE Trans. Microw. Theory Tech.* **68**, 3834–3851 (2020).

19. P. Nazari, S. Jafarlou, P. Heydari, A CMOS two-element 170-GHz fundamental-frequency transmitter with direct RF-8PSK modulation. *IEEE J. Solid-State Circuits* **55**, 282–297 (2020).

20. H. Hamada, T. Tsutsumi, H. Matsuzaki, T. Fujimura, I. Abdo, A. Shirane, K. Okada, G. Itami, H.-J. Song, H. Sugiyama, H. Nosaka, 300-GHz-band 120-Gb/s wireless front-end based on InP-HEMT PAs and mixers. *IEEE J. Solid-State Circuits* **55**, 2316–2335 (2020).

21. T. J. Cui, M. Q. Qi, X. Wan, J. Zhao, Q. Cheng, Coding metamaterials, digital metamaterials, and programmable metamaterials. *Light Sci. Appl.* **3**, e218–e218 (2014).

22. L. Zhang, X. Q. Chen, S. Liu, Q. Zhang, J. Zhao, J. Y. Dai, G. D. Bai, X. Wan, Q. Cheng, G. Castaldi, V. Galdi, T. J. Cui, Space-time-coding digital metasurfaces. *Nat. Commun.* **9**, 4334 (2018).

23. T. J. Cui, S. Liu, G. D. Bai, Q. Ma, Direct transmission of digital message via programmable coding metasurface. *Research* **2019**, 2584509 (2019).

24. J. Zhao, X. Yang, J. Y. Dai, Q. Cheng, X. Li, N. H. Qi, J. C. Ke, G. D. Bai, S. Liu, S. Jin, A. Alù, T. J. Cui, Programmable time-domain digital coding metasurface for nonlinear harmonic manipulation and new wireless communication systems. *Natl. Sci. Rev.* **6**, 231–238 (2019).

25. T. J. Cui, S. Liu, L. Zhang, Information metamaterials and metasurfaces. *J. Mater. Chem. C* **5**, 3644–3668 (2017).

26. T. J. Cui, L. Li, S. Liu, Q. Ma, L. Zhang, X. Wan, W. X. Jiang, Q. Cheng, Information metamaterial systems. *iScience* **23**, 101403 (2020).

27. J. Y. Dai, J. Zhao, Q. Cheng, T. J. Cui, Independent control of harmonic amplitudes and phases via a time-domain digital coding metasurface. *Light Sci. Appl.* **7**, 90 (2018).

28. T. J. Cui, S. Liu, L.-L. Li, Information entropy of coding metasurface. *Light Sci. Appl.* **5**, e16172–e16172 (2016).

29. M. Z. Chen, W. Tang, J. Y. Dai, J. C. Ke, L. Zhang, C. Zhang, J. Yang, L. Li, Q. Cheng, S. Jin, T. J. Cui, Accurate and broadband manipulations of harmonic amplitudes and phases to reach 256QAM millimeter-wave wireless communications by time-domain digital coding metasurface. *Natl. Sci. Rev.* **9**, nwab134 (2022).

30. J. Y. Dai, W. Tang, M. Z. Chen, C. H. Chan, Q. Cheng, S. Jin, T. J. Cui, Wireless communication based on information metasurfaces. *IEEE Trans. Microw. Theory Tech.* **69**, 1493–1510 (2021).

31. J. Y. Dai, W. K. Tang, J. Zhao, X. Li, Q. Cheng, J. C. Ke, M. Z. Chen, S. Jin, T. J. Cui, Wireless communications through a simplified architecture based on time-domain digital coding metasurface. *Adv. Mater. Technol.* **4**, 1900044 (2019).

32. Q. Cheng, L. Zhang, J. Y. Dai, W. Tang, J. C. Ke, S. Liu, J. C. Liang, S. Jin, T. J. Cui, Reconfigurable intelligent surfaces: Simplified-architecture transmitters—From theory to implementations. *Proc. IEEE* **110**, 1266–1289 (2022).

33. L. Zhang, M. Z. Chen, W. Tang, J. Y. Dai, L. Miao, X. Y. Zhou, S. Jin, Q. Cheng, T. J. Cui, A wireless communication scheme based on space- and frequency-division multiplexing using digital metasurfaces. *Nat. Electron.* **4**, 218–227 (2021).

34. S. R. Wang, J. Y. Dai, Q. Y. Zhou, J. C. Ke, Q. Cheng, T. J. Cui, Manipulations of multi-frequency waves and signals via multi-partition asynchronous space-time-coding digital metasurface. *Nat. Commun.* **14**, 5377 (2023).

35. C. Liaskos, S. Nie, A. Tsioliaridou, A. Pitsillides, S. Ioannidis, I. Akyildiz, A new wireless communication paradigm through software-controlled metasurfaces. *IEEE Commun. Mag.* **56**, 162–169 (2018).

36. J. Zhao, X Yang, J. Y. Dai, Q. Cheng, X. Li, N. H. Qi, J. C. Ke, G. D. Bai, S. Liu, S. Jin, A. Alù, T. J. Cui, Programmable time-domain digital-coding metasurface for non-linear harmonic manipulation and new wireless communication systems, *Natl. Sci. Rev.* **6**, 231–238 (2019).

37. Y. Hadad, D. L. Sounas, A. Alu, Space-time gradient metasurfaces. *Phys. Rev. B* **92**, 100304 (2015).

38. J. Y. Dai, W. Tang, L. X. Yang, X. Li, M. Z. Chen, J. C. Ke, Q. Cheng, S. Jin, T. J. Cui, Realization of multi-modulation schemes for wireless communication by time-domain digital coding metasurface. *IEEE Trans. Antennas Propag.* **68**, 1618–1627 (2020)

39. J. Y. Dai, L. X. Yang, J. C. Ke, M. Z. Chen, W. K. Tang, X. Li, M. Chen, Z. H. Wu, Q. Cheng, S. Jin, T. J. Cui. High-efficiency synthesizer for spatial waves based on space-time-coding digital metasurface. *Laser Photonics Rev.* **14**, 1900133 (2020).

40. L. Zhang, Z. X. Wang, R. W. Shao, J. L. Shen, X. Q. Chen, X. Wan, Q. Cheng, T. J. Cui, Dynamically realizing arbitrary multi-bit programmable phases using a 2-bit time-domain coding metasurface. *IEEE Trans. Antennas Propag.* **68**, 2984–2992 (2020).

41. S. Liu, T. J. Cui. Concepts, working principles, and applications of coding and programmable metamaterials. *Adv. Opt. Mater.* **5**, 1700624 (2017).

42. Y. Zhang, K. Ding, H. Zeng, W. Kou, T. Zhou, H. Zhou, S. Gong, T. Zhang, L. Wang, S. Liang, F. Lan, Y. Dong, Z. Feng, Y. Gong, Z. Yang, D. M. Mittleman, Ultrafast modulation of terahertz waves using on-chip dual-layer near-field coupling. *Optica* **9**, 1268–1275 (2022)

43. N. G. Wright, A. G. O'Neill, C. M. Johnson, High temperature/high power Schottky diodes. *Mater. Sci. Eng. B* **46**, 57–60 (1997).

44. L. E. Wernersson, M. Borgström, B. Gustafson, A. Gustafsson, I. Pietzonka, M. E. Pistol, T. Sass, W. Seifert, L. Samuelson, Metalorganic vapor phase epitaxy-grown GaP/GaAs/GaP and GaAsP/GaAs/GaAsP n-type resonant tunnelling diodes. *Appl. Phys. Lett.* **80**, 1841–1843 (2002).

45. S. Abadal, C. Han, J. M. Jornet, Wave propagation and channel modeling in chip-scale wireless communications: A survey from millimeter-wave to terahertz and optics. *IEEE Access* **8**, 278–293 (2020).

46. M. F. Imani, S. Abadal, P. Del Hougne, Metasurface-programmable wireless network-on-chip. *Adv. Sci.* **9**, e2201458 (2022).

47. J. Tapie, H. Prod'homme, M. F. Imani, P. del Hougne, Systematic physics-compliant analysis of over-the-air channel equalization in RIS-parametrized wireless networks-on-chip. arXiv:2310.16195 [physics.app-ph] (2023).

48. R. Schmogrow, B. Nebendahl, M. Winter, A. Josten, D. Hillerkuss, S. Koenig, J. Meyer, M. Dreschman, M. Huebner, C. Koos, J. Becker, W. Freude, J. Leuthold, Error vector magnitude as a performance measure for advanced modulation formats. *IEEE Photonics Technol. Lett.* **24**, 61–63 (2011).

49. I. Dan, G. Ducournau, S. Hisatake, P. Szriftgiser, R. P. Braun, I. Kallfass. A superheterodyne 300 GHz wireless link for ultra-fast terahertz communication systems. *Int. J. Microw. Wirel. Technol.* **12**, 578–587 (2020).

50. D. Wrana, L. John, B. Schoch, S. Wagner, I. Kallfass. Sensitivity analysis of a 280–312 GHz superheterodyne terahertz link targeting IEEE802.15.3d applications. *IEEE Trans. Terahertz Sci. Technol.* **12**, 325–333 (2022).

51. A. Standaert, P. Reynaert. A 390-GHz outphasing transmitter in 28-nm CMOS. *IEEE J. Solid-State Circuits* **55**, 2703–2713 (2020).

52. I. Abdo, T. Fujimura, T. Miura, K. K. Tokgoz, H. Hamada, H. Nosaka, A. Shirane, K. Okada. A 300GHz wireless transceiver in 65nm CMOS for IEEE802.15.3d using push-push subharmonic mixer, paper presented at the *2020 IEEE/MTT-S International Microwave Symposium (IMS)*, Los Angeles, CA, USA, 4 to 6 August 2020.

53. S. Jia, M. C. Lo, L. Zhang, O. Ozolins, A. Udalcovs, D. Kong, X. D. Pang, R. Guzman, X. B. Yu, S. L. Xiao, S. Popov, J. J. Chen, G. Carpintero, T. Morioka, H. Hu, L. K. Oxenløwe. Integrated dual-laser photonic chip for high-purity carrier generation enabling ultrafast terahertz wireless communications. *Nat. Commun.* **13**, 1388 (2022).

54. J. Webber, A. Oshiro, S. Iwamatsu, Y. Nishida, M. Fujita, T. Nagatsuma. 48-Gbit/s 8K video-transmission using resonant tunnelling diodes in 300-GHz band. *Electron. Lett.* **57**, 668–669 (2021).
